# A solitary ulcer on the tongue

**DOI:** 10.1007/s10354-025-01102-x

**Published:** 2025-08-21

**Authors:** Annalina Meyka, Eva Schadelbauer, Birger Kränke, Birgit Sadoghi

**Affiliations:** Department of Dermatology and Venereology, Medical University of Graz, Auenbruggerplatz 8, 8036 Graz, Austria

**Keywords:** Syphilis, Oropharyngeal ulcer, Oral chancre, *Treponema pallidum*, Sexually transmitted infection, Syphilis, Oropharyngeale Ulzeration, Oraler Schanker, Treponema pallidum, Sexuell übertragbare Infektion

## Abstract

Syphilis is a systemic sexually transmitted infection caused by the invasive bacterium *Treponema pallidum*. The diagnosis remains challenging since the disease is labeled as “the great imitator” due to its broad clinical and histopathological appearances. Due to a significant rise in its incidence rates, syphilis should always be considered as a differential diagnosis of any painless, newly developed, oropharyngeal ulcerated lesion. This case demonstrates the rare clinical appearance of a chancre on the tongue as the primary clinical sign of syphilis.

## Abstract (de)

Syphilis ist eine systemische, sexuell übertragbare Infektion, hervorgerufen durch das invasive Bakterium *Treponema pallidum*. Die Diagnosestellung gestaltet sich weiterhin herausfordernd, da die Erkrankung aufgrund ihres breiten Spektrums an klinischen und histopathologischen Erscheinungsformen als „der große Imitator“ bezeichnet wird. Angesichts des signifikanten Anstiegs der Inzidenzraten sollte Syphilis als Differenzialdiagnose bei jeder schmerzlosen, neu aufgetretenen ulzerierten Läsion im Oropharynx in Betracht gezogen werden. Dieser Fallbericht zeigt das seltene klinische Auftreten eines Schankers an der Zunge als primäres Anzeichen einer Syphilisinfektion.

## Case report

**Fig. 1:**
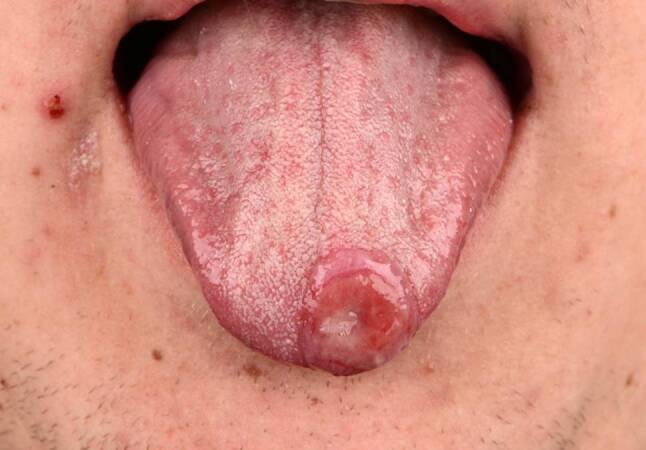
Solitary thumb-nail-sized ulcer at the tip of the tongue

An 18-year-old white male suffered from a solitary, about 1.5 cm in diameter, partly fibrin-covered, slightly painful, sharply demarcated ulcer on the tip of the tongue persisting for some weeks (Fig. 1), which led him to present to his otorhinolaryngologist. He had an unremarkable medical history with no known pre-existing conditions, did not take any medication, and had not experienced similar episodes before. The last unprotected heterosexual intercourse with his committed partner was 1.5 months ago; he denied any random sexual contact. Upon inquiry, the patient reported that his sexual activity over the preceding 6‑month period was exclusively limited to intercourse with his current partner.

Despite a discrete pharyngeal enanthema, submandibular lymphadenopathy was present. A punch biopsy of the lesion revealed capillary-rich granulation tissue with superficial ulceration, without hints of malignancy. Due to persistence of the ulcer, the patient was referred to the Department of Dermatology.

## What is your diagnosis?

A. Herpetic gingivostomatitis
B. Behcet disease
C. Primary syphilis
D. Eosinophilic ulcer

## Diagnosis

### Primary syphilis

This case demonstrates the characteristic clinical appearance of a chancre, the primary clinical sign of syphilis; however, it is rarely found in this classic appearance and seldom in the intraoral region. Serology confirmed syphilis (rapid plasma regain [RPR] test 1:2; *Treponema pallidum* particle agglutination assay [TPPA] > 1:1280; syphilis antibody screening test IgG, IgM, IgA version Ultra Diapro® reactive; and treponemal membrane protein A enzyme-linked immunosorbent assay IgM reactive). Additional sexually transmitted infection (STI) testing included serological tests for hepatitis B, hepatitis C, and HIV, which were all negative.

## Discussion

Syphilis is a systemic sexually transmitted infection caused by the invasive bacterium *Treponema pallidum* [1, 2]. Over the past years the number of syphilis cases has risen steadily [1–3]. The resurgence of syphilis can likely be attributed to several factors, including the growing use of dating applications, an increase in casual sexual encounters, and disruptions in STI surveillance and screening during the COVID-19 pandemic. Furthermore, the overall rise in STIs is closely associated with the introduction of highly effective HIV PrEP. However, due to its proven efficacy, many individuals are opting to forgo condom use [4]. After an incubation period of up to 90 days, syphilis manifests as an ulcer, also known as a chancre, occurring at the site of entry of the pathogen, mostly in the genital area and seldom extragenitally [2, 3, 5–7]. The ulcer is typically painless, with sharply defined firm, often elevated, margins and an infiltrated base with a slightly depressed center. About 4–12% of patients with primary syphilis suffer from oral chancres, often associated with lymphadenopathy [6, 7]. The primary syphilitic oral locations seem to be the vermilion and mucosa of the lips, along with the dorsum and lateral border of the tongue [6–9].

Oral manifestations, however, can appear at any stage and are often prevalent in the highly infectious secondary stage; the variety of their clinical manifestation is broad [1, 2, 8–11]. Most frequently, syphilitic lesions present themselves as enanthematous, mucous patches, papules, or plaques or as erosions with slightly elevated borders [2, 8–11]. Moreover, syphilitic tonsillitis (angina specifica) is a possible clinical manifestation.

Diagnosing syphilis remains challenging, and the disease has been labeled as “the great imitator” due to its various clinical and histopathological appearances imitating several diseases [2, 10, 11]. Regarding the oropharyngeal region, differential diagnoses like herpes labialis, pyogenic granuloma, aphthous or herpetic stomatitis, lichen planus, pemphigus, Behcet disease, or malignant neoplasms must be kept in mind [5].

While the presence of plasma cells, especially within or around blood vessels and at the dermoepidermal junction, can suggest syphilis (among other conditions such as Lyme disease, leishmaniasis, lymphoma, and lupus erythematosus), a definitive histological diagnosis of syphilis can only be made through *Treponema pallidum* immunohistochemical staining, which allows for the visualization of red-stained spirochetes. Beyond this specific finding, syphilis lacks pathognomonic histopathological features, making its definitive diagnosis challenging through routine histological examination alone [12]. Histology is not the method of choice for diagnosis. Direct detection of *Treponema pallidum* can be performed via dark-field microscopy at the anogenital region but not at the intraoral site, due to commensal bacteria. Polymerase chain reaction (PCR) testing can be done at any site [1, 2]. In our case, PCR testing for *Treponema pallidum* was not yet available, and the diagnosis was based on the typical clinical appearance and serology. Serological testing in syphilis is inevitable, as it is the only method to evaluate therapeutic efficacy. Given the high contagiousness of the first and second stages of the disease, early recognition of clinical features is crucial, as misdiagnosis may lead to serious consequences and relevant morbidity [1–3]. Treatment is performed with a single injection of 2.4 million IE benzathine penicillin intramuscularly for early syphilis and three injections every other week for late syphilis [1, 2].

## Outcome

First-line therapy with a single-dose of 2.4 million units of intramuscular long-acting benzathine benzylpenicillin (BPG) was well tolerated and led to rapid remission of the lesion. Serological activity parameters (PRP) declined within 3 months. The patient denied any previous syphilitic infection. Additional STI testing included serological tests for hepatitis B, hepatitis C, and HIV, which were all negative. The patient’s partner, who had been informed of the diagnosis by the patient himself, subsequently presented at our outpatient clinic. During the visit, the partner was provided with updated information regarding the current situation and, following comprehensive counselling, received immediate prophylactic therapy, as recommended in current guidelines. Due to the high rise in incidence, syphilis should always be considered in the differential diagnosis of any painless, newly developed, oropharyngeal ulcerated lesions, regardless of a patient’s medical history or age.
